# Co-densification of rice straw and cow dung in different food-to-microorganism ratios for biogas production

**DOI:** 10.1038/s41598-024-52122-3

**Published:** 2024-03-11

**Authors:** Prakash Singh, Pallavi Dogra, Induchoodan TG, Ajay S. Kalamdhad

**Affiliations:** 1 School of Agro and Rural Technology, Indian Institute of Technology Guwahati, Guwahati, Assam 781039 India; 2 Department of Civil Engineering, Indian Institute of Technology Guwahati, Guwahati, Assam 781039 India

**Keywords:** Renewable energy, Bioenergy

## Abstract

Agricultural residues such as rice straw (RS) are desirable raw materials for biogas generation. However, the recalcitrant nature of RS hinders biogas production, and its low bulk density increases storage space requirements, transportation needs, and overall costs. These challenges could be resolved by pretreatment and pelletization. In this study, various thermal pretreatments were performed, and the best conditions (temperature and time) were identified. Also, rice straw and cow dung pellets (RCP) at different food-to-microorganism (F/M) ratios (0.5–2.5) were prepared. Parameters such as bulk density, moisture absorption, and drop shatter tests were conducted to evaluate the physical properties. Finally, the biochemical methane potential (BMP) study of the best RCP with varying total solids (TS: 4–12%) content was investigated. The results indicate that hot air oven pretreatment (for 60 min at 120 °C) resulted in maximum solubilization. In physical characteristics, RCP with an F/M ratio of 2.5 pellets was ideal. The bulk density of RCP 2.5 was found to be around 25 times that of the raw. Also, the TS 8% yielded maximum biomethane (279 mL/g-VS_consumed_) as compared to other TS contents. Overall, this study will propel the growth of bioenergy while simultaneously tackling the pressing issues related to RS management.

## Introduction

The biological conversion of organic matter via anaerobic digestion produces methane-rich biogas. In addition, digestate is produced, which is a desirable organic fertilizer in agriculture. Production of biogas from organic waste reduces environmental challenges such as groundwater and soil contamination, and emissions of air pollutants^1^. There are various types of substrates from which biogas can be produced. Among these, animal dung and agricultural residues, due to their abundance, are considered viable organic materials for the production of biogas, which has been widely used or implemented around the world due to its various energy and environmental advantages^2^.

More than half of India’s population is dependent on agriculture for its livelihood, using 179.8 million hectares of agricultural land^3^. According to estimates, India has 708 million metric tonnes of lignocellulosic agro-residue biomass, of which a surplus of 209 million metric tonnes (or 30% of the total) can be used to produce energy sustainably^4^. India has considerable potential (around 75 billion m^3^/year) for biogas generation. The findings suggest that agricultural residue has a maximum contribution (39 billion m^3^/year, 52%) to total biogas potential^4^. At the individual crop level, the potential of rice straw was found to be second highest at 9 billion m^3^/year^4^.

Agricultural residues and its potential to produce bioenergy, including through co-digestion with other organic materials, have recently drawn more attention^5^. However, the bulk density of most agricultural residues is lower, which raises the price of storage and transportation. Compacting increases density and other properties while decreasing volume, which also reduces storage requirements, transport requirements, and overall costs^6^.

The process of biomass densification, which involves the pelletization of agricultural waste to increase compositional homogeneity, has been identified as a potentially viable alternative for improving physicochemical properties for biomethane yield and residue storage^7,8^. It is essential to note that the densification of mono-straw may result in a significant increase in capital and operating costs, ranging from 3 to 35%, due to its high energy consumption, particularly in the case of rice/wheat straw, which lacks the efficient binding capability of lignocellulosic components^9^.

To enhance the binding between inter-particles and reduce the additional energy requirement, co-densification has been developed, in which different materials are utilized as binders, resulting in improved performance^10^. Different materials, such as molasses combined with crude glycerol^11^, soaked brown sugar water^12^, plastic garbage^13^, and pyrolysis bio-oil^14^, have demonstrated the potential to enhance strength and densification efficiency. These materials may not be the most convenient binders in rural areas due to its inherent viscous nature and/or limited availability at the household level. Consequently, few examinations have focused on the utilization of animal dung as a prospective substitute^15^. However, in this particular study, the pellets were produced for combustion. It is also important to note that animal dung, especially cow dung (CD), is often used in biochemical methane potential (BMP) setups as a source of inoculum. Each anaerobe has a unique ability to break down substrate for that ideal situation, which must be maintained by its buffering ability, which can only be maintained in a particular food-to-microorganism (F/M) ratio^16^. From a co-densification perspective, in previous studies, the combination of cow dung and wheat straw increased the bulk density and increased the calorific value for solid biofuel production^15^. In addition, the combined digestion of manure and straw, with a balanced carbon and nitrogen ratio (C:N) of 20–30, may produce a synergistic outcome that increases biogas yield^17^. The C:N ratio of crop straw is usually 50–70, while that of animal manure is 10–20^18,19^. Despite this, co-densification's impact has been largely overlooked.

The available literature on the densification of agricultural residue^8,20,21^ for biogas generation has not taken the concept of the F/M ratio into account. Also, the pellets’ physical properties were not evaluated, and optimization of the total solids (TS) content was not performed. Thus, this study aims to bridge a significant gap in the existing literature by exploring a new approach to the co-densification of rice straw (RS) and cow dung (CD) for biomethane production. By leveraging the advantages of pelletization in increasing the biomethane yield, this study incorporated the food-to-microorganism ratio during the co-densification process.

The novelty of this work includes the biomass co-densification of RS and CD. Furthermore, this study also takes the concept of the F/M ratio from BMP studies and introduces it in the co-densification stage itself. The present study endeavours to assess the viability of utilizing hybrid pellets comprising rice straw and cow dung (RCP) as a versatile raw material for biogas generation, thus optimizing the total solids (TS) content. The aim of the study is to enhance the biomethane potential of the RS using pretreatment methods such as hot air ovens, autoclaves, hot water baths, and microwave oven pretreatments for accelerated hydrolysis in anaerobic digestion. Also, blended pellets of the pretreated (i.e., best pretreatment) rice straw and cow dung (RCP) were prepared in different F/M ratios (0.5 to 2.5), and physical characteristics tests (bulk density, water penetration, and friability) were conducted to assess its suitability for transportation and storage purposes. Finally, the biogas production of the best F/M ratio RCP pellets (i.e., physical characteristics) was determined by optimizing total solids (TS) content. This research aims to advance sustainable bioenergy production while addressing critical agricultural waste management challenges.

## Materials and methods

### Materials

The RS residue and fresh CD were obtained from a field at Guwahati, in Assam (India), and were reduced in size using a shredding machine. The substrate's surface area, porosity, and potential degradability are all increased by particle size reduction. Table 1 shows the initial characterization of RS and CD used in this study. The moisture content (MC), total solids (TS), and volatile solids (VS) are determined using the standard method by the American Public Health Authority^22^. For the calculation of MC and TS, a known weight of substrates was taken in a crucible. The sample was dried at a temperature of 105 °C for 24 h. The final weight was noted, and the TS% was evaluated as the percentage of the final sample weight divided by the initial sample weight. The fixed solids were measured using a muffle furnace, where a known weight of a sample is kept at 550 °C for 2 h before measuring the residue. The volatile solids were calculated by subtracting the fixed solids from the TS. The sample’s pH was measured using a portable pH meter. The ratio of volatile solids to total solids (VS/TS) can be used to express the variation of volatile matter in dry material per unit. The high percentage of VS/TS indicates a significant amount of organic content that can be effectively utilized. Volatile matter represents the feedstock's sole component that microorganisms can convert into biogas.

**Table 1:** Initial characterization of rice straw and cow dung.

	Moisture content (MC%)	Volatile Solids (VS%)	Total solids (TS%)	VS(%TS)	pH
Rice straw	11.58 ± 1.07	69.20 ± 2.86	88.42 ± 4.63	78.26	6.9 ± 0.3
Cow dung	77.25 ± 0.7	11.64 ± 0.8	22.75 ± 0.3	52.28 ± 1.2	7.57 ± 0.4

± standard deviation.

### Pretreatment study

To increase the degradability of RS and hasten the anaerobic digestion process, biomass with a high lignin content must be pretreated^23^.

#### Sample preparation

The RS samples were manually cleaned, ground, and sieved to remove particulate matter. Samples were made by combining 5 g of ground RS with 50 ml of distilled water in a conical flask (glassware) separately to create the water-soluble extract. Substrate-filled conical flasks were then mechanically shaken for 2 h at 100 rpm to ensure consistency in the sample.

#### Thermal pretreatment

This study compared the effects of various thermal pretreatment methods with various temperature and time conditions on the degradability and solubility of RS^24–26^.

##### Hot air oven pretreatment

For pretreatment, RS samples were prepared in sealed conical flasks and left to stand in a hot air oven (Fisher Scientific, Isotemp 637G Oven, USA), which transfers heat energy throughout the sample using the conduction and convection principles. In a prior study, Kainthola et al.^26^ suggested pretreatment temperatures of 60, 70, 80, 90, 100, 110, and 120 °C for 45 min as exposure time for rice straw (lignocellulosic material). The best temperature was chosen based on the ideal conditions discovered during RS's temperature study. Additionally, a trial was conducted to determine the best exposure time out of a series of exposure times, including 30, 60, 90, and 120 min for each of the selected temperatures.

##### Hot water bath pretreatment

A hot water bath (Fisher Scientific, Isotemp 3016H, USA) is used to hydrate the intricate structure of RS while heating sealed conical flasks containing RS samples. Thus, this process is also called hydrothermolysis. For 30, 60, 90, and 120 min, RS samples were exposed to pretreatment temperatures of 60, 70, 80, 90, and 95°C^27^. Based on the maximum solubilization expressed as soluble chemical oxygen demand (sCOD), the best pretreatment temperature was chosen.

##### Autoclave pretreatment

Pretreatment involved placing conical flasks of RS in an autoclave where water vapour (steam) transfers heat through conduction and convection to rupture the RS matrix, a process known as autohydrolysis. The pretreatment temperature was 120 °C for 10, 20, 30, 45, and 60 min exposure durations, with the optimal condition being the time with the highest solubilization (sCOD)^26,27^.

##### Microwave oven pretreatment

In the microwave, energized electrons or photons transferred heat across sealed conical flasks with RS samples to break down RS's complicated structure. The sample absorbs energy continuously as it rotates in the oven's electromagnetic field. Pretreatment temperatures of 100, 140, 160, 180, 200, and 220 °C for 2, 3, 4, and 5 min were used, with the optimal temperature achieving the highest sCOD^28^.

### Pelletization

**Figure 1:**
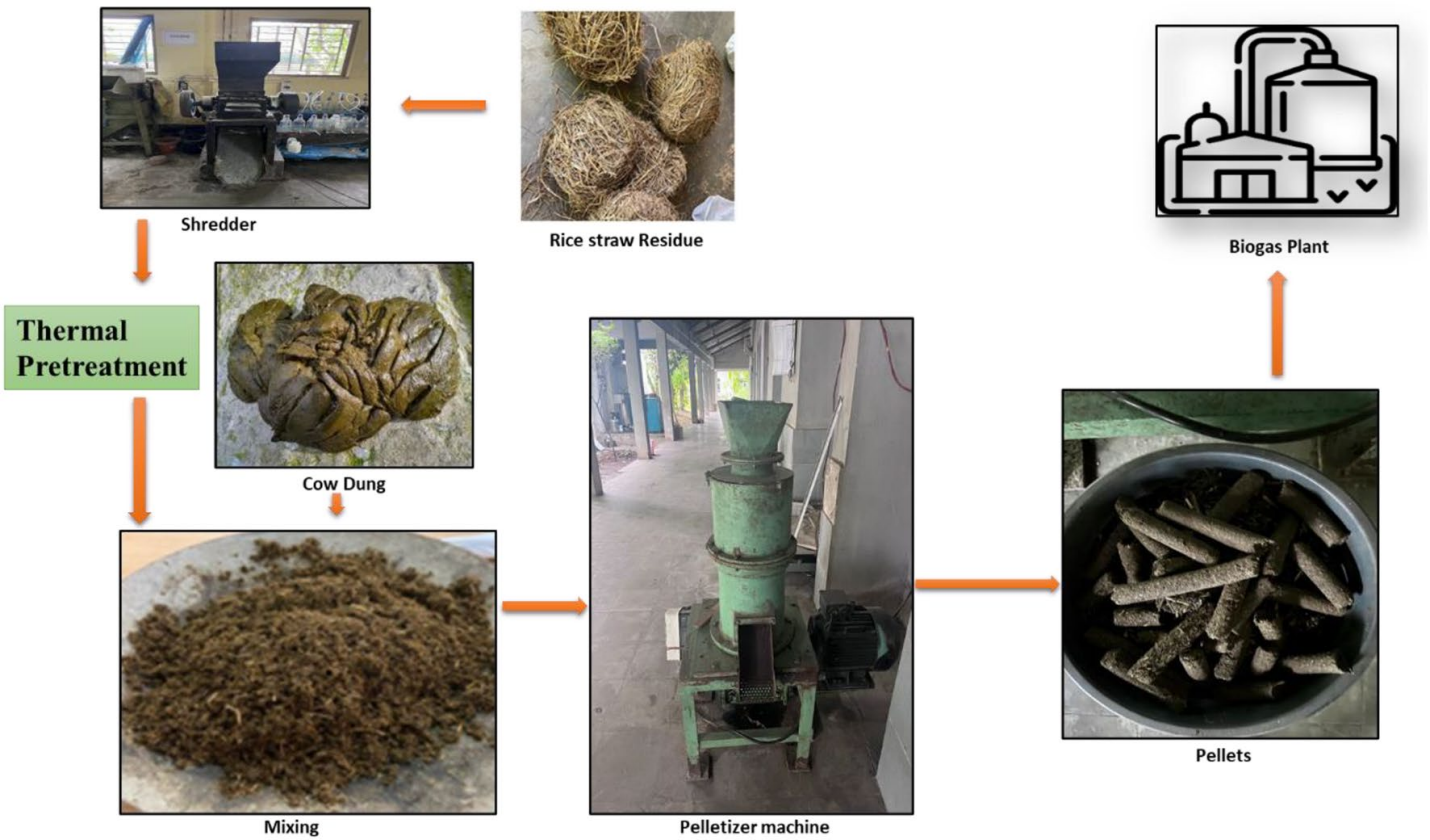
Pelletization process.

The RS was subjected to natural drying to reduce its moisture content. Once dried, the RS was mechanically chopped using a shredder, ensuring a consistent particle size. The residue was then subjected to the best pretreatment (i.e. hot air oven) to enhance its biomethane potential. The cow dung, known for its active microbial community and binding properties, was selected as the binder material (or co-densification material) and mixed with the chopped RS at varying (F/M) ratios, as shown in Table 2. The (F/M) ratio is important in all batch anaerobic digestion processes and VS degradation^16^. The quantity of crop residue and cow dung utilized was determined based on the calculated VS content. For RS, in previous studies, different F/M ratios in the range of 0.5–2.5 were evaluated for BMP test^26,29^. Hence in this study, pellets were fabricated in these ratios. To facilitate the pelletization process, a roller press pelletizer was used in the experiment. Before pelletization, the blended mixture was conditioned by adding 3%–5% distilled water (w/w) to enhance its workability. Subsequently, the mixed biomass was fed into the pelletizer and passed through the equipment twice to ensure the formation of uniform rice straw and cow dung pellets (RCP) (Fig. 1).

**Table 2:** Crop residue and cow dung mixing ratio at varying F/M ratios.

Crop residue	F/M ratio	Naturally dried crop residue (CR) (g)	Cow dung (g)	Ratio CR: CD
Rice straw and Cow dung pellets (RCP)	0.5	43.3	500	1:12
	1.0	86.7	500	1:6
	1.5	130	500	1:4
	2.0	173.4	500	1:3
	2.5	216.7	500	1:2

Following the pelletization process, the freshly formed RCP was manually spread on the cement floor and covered with a plastic film, allowing for adequate ventilation. These pellets were then placed in a solar greenhouse for five days. During this time, the pellets underwent a cooling, settling, and drying process. To assess the quality and properties of the pellets, standard procedures were used to determine its bulk density.

In addition to determining the bulk density, further assessments were conducted to evaluate their physical characteristics. Water penetration and friability tests were performed to gain insights into the structural properties of the pellets. These tests aimed to assess the ability of the pellets to resist fragmentation or breakage.

#### Physical characterization of pellets

The physical characteristics of the pellets were examined to assess their suitability for transportation and storage purposes. Three key characterizations were conducted, namely bulk density, water penetration, and friability, each playing a crucial role in evaluating the feasibility and performance of the pellets.

To determine the bulk density of the pellets, the American Society of Agricultural and Biological Engineers (ASABE) Standard S269.4 (2007) for cubes, pellets, and crumbles was followed^30^. The pellets were carefully poured into a cylindrical container from a specific height to ensure smooth flow until the container reached its maximum capacity. The top of the container was then levelled by using a straight edge to eliminate any extra material. By subtracting the weight of the empty container from the combined weight of the pellets and container, the net weight of the pellets was obtained. The bulk density was calculated by dividing the mass by the volume of the container. For each type of pellet, the presented data are the average of three measurements.

1 $$\mathrm{Bulk \,Density}=\frac{{\text{m}}}{{\text{V}}}$$

Moisture content is a critical factor that can affect biomass pellets' mechanical properties and durability during storage. To assess the water penetration characteristics, the percentage of water absorbed by pellets was measured^31^. The dried sample was initially weighed and recorded w_1_, after which it was submerged in water at a controlled temperature of 23 °C for two hours. Subsequently, the sample was weighed again to determine the amount of water absorbed as w_2_. The water penetration was calculated using a designated formula designed for this purpose.

2 $$\mathrm{Water\, Penetration}=\frac{{{\text{w}}}_{2}-{{\text{w}}}_{1}}{{{\text{w}}}_{1}}\mathrm{ x }100$$

The friability test also referred to as the drop shatter test, is used to evaluate the resilience and integrity of crop residue pellets in terms of breakage. Firstly, the sample of crop residue pellets is weighed using a precision balance, and the initial weight is duly recorded. Subsequently, the pellets are subjected to two consecutive drops from a height of 6 feet^32^. After the pellets had been dropped, the shattered fragments were carefully collected and removed from the collection pan or tray. The gathered fragments are then weighed using the precision balance. The friability index, or drop-shatter index can be computed by using the following formula:

3 $$\mathrm{Friability\, Index}=\frac{\mathrm{Weight\, of\, Shattered\, Fragments }}{\mathrm{Initial\, Weight\, of\, Pellets}}\mathrm{ x }100$$

Using the friability test, the resistance of crop residue pellets to breakage and durability was determined, which is crucial for transportation, handling, and storage purposes.

These tests played a crucial role in determining the optimal feed-to-microorganism ratio for the pellets based on their physical attributes. This was further used to optimize the total solid content based on the optimized F/M ratio. Such detailed evaluations allowed for informed decisions regarding the suitability and effectiveness of the crop residue pellets in transportation and storage.

#### Biochemical methane potential of RCP based on total solids content

The BMP reactor setup was carried out in 1L glass bottles (batch reactor) closed with rubber corks, and at the end, all the anaerobic reactors were coupled to an aspirator bottle with a 1.5 N sodium hydroxide (NaOH) solution to scrub the carbon dioxide (CO_2_) and other trace amounts of gases (N_2_, NH_3_, etc.) as explained in previous literature^33–35^. The quantification of biomethane (CH_4_) produced is calculated by the liquid (1.5 N NaOH) displacement method^16,36^. The test was performed in ambient (room) conditions. The temperature during the period was between 30 to 38 °C. The best identified F/M ratio RCP was studied with different total solid contents (4%, 6%, 8%, 10%, and 12%). The RCP was added in 1L glass bottles using different total solid content with distilled water, as outlined in Table 3. The methane produced was measured daily using the liquid displacement method, and other parameters like MC, VS, sCOD, and VFA were measured on a weekly basis. The MC and VS were calculated as mentioned in Section "Materials". The sCOD was determined according to the standard method outlined in APHA^22^. The VFA was quantified using the titration method based on pH^37^.

**Table 3:** Total solid content for BMP setup.

Pellets	Total solid (%)	Water (mL)	Total solid (g)	Pellets (g)	Feed dilution ratio FDR
Rice straw and Cow dung pellets (RCP)	4%	700	28	29.1	1:24
	6%	700	42	43.6	1:16
	8%	700	56	58.2	1:12
	10%	700	70	72.7	1:10
	12%	700	84	87.3	1:8

4 $$\mathrm{Feed \,Dilution\, Ratio }({\text{FDR}})=\frac{\mathrm{Mass \,of \,feed \,biomass}}{\mathrm{Mass\, of \,water}}$$

### Instrumentation analysis

Field Emission Scanning Electron Microscopy (FESEM), was used to examine the structural changes during the pretreatment of crop residue. FESEM provided surface imaging of the substrate, allowing for the observation of microscale structural changes. Parameters such as an accelerating voltage of 20 kV and an energy filter setting of 130 eV were maintained during FESEM analysis. Chemical changes during the pretreatment process were observed with Fourier transform infrared (FTIR) (frequency range 4000 to 500 cm^−1^).

## Results and discussions

### Pretreatment study

The effects of various thermal pretreatment methods (hot air oven, hot water bath, microwave, and autoclave) were compared using the solubilization of RS. To demonstrate the suitability and relative usefulness of the various thermal pretreatment techniques for increased methane production, the hydrolysis behavior of raw and pretreated residues was examined. Since the RS is solubilized during the pretreatment, the overall time taken to complete the anaerobic digestion of RS is also reduced. It increases the biomass's accessible surface area and increases its accessibility to hydrolytic enzymes. The relationship between compositional changes and the ideal residence time and temperature was another area of emphasis in this study.

**Figure 2:**
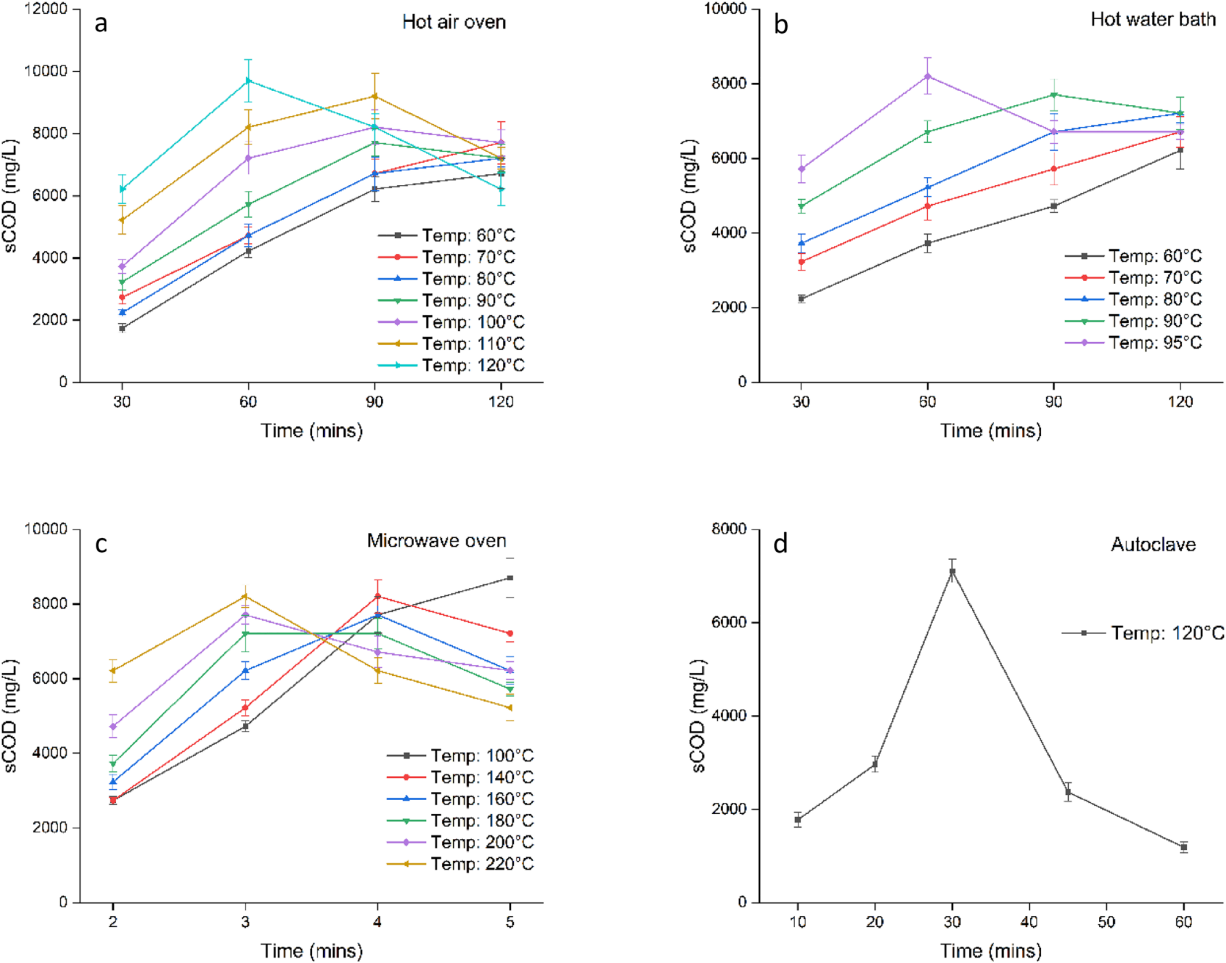
Pretreatment study for rice straw. **a** Hot Air Oven **b** Hot water bath **c** Microwave Oven **d** Autoclave.

Figure 2 depicts the temperature and temporal pretreatment studies of rice straw, respectively, where the substrate was allowed to pretreat thermally. The percentage increment in sCOD for the pretreated biomass compared to the untreated biomass delineates the degree of solubilization. Among the four thermal pretreatment techniques, the hot air oven pretreatment showed maximum solubilization with an increment of 67.4% in sCOD, followed by microwave (50.2%), hot water bath (41.3%), and autoclave (22.4%). The differences may be due to variations in evaporation between different equipment. Thermal pretreatment shortens hydrolysis in two ways. First, water present in the biomass, with the help of additional heat, destroys the organic compound's hydrogen bonds that hold the crystalline lignocellulosic structure. Second, it depolymerizes cellulose and hemicellulose into short-chain monomers^38,39^. In the pretreatment study, hot air oven pretreatment was obtained as the ideal pretreatment technique for RS with operating conditons of 120 °C for 60 min with a sCOD value of 9699 mg/L. This study's findings are comparable with previous studies, where, after pretreatment, an increase in sCOD solubilization of 66.6% from rice straw^40^ and 55.5% from water hyanicth^27^ was observed, respectively. Also, for lignocellulosic biomass such as water hyacinth and pulp and paper mill sludge, a hot air oven was identified as the best treatment method^27,28,38^.

#### Effect of pretreatment on the instrumental characterization of RS

##### FESEM

**Figure 3:**
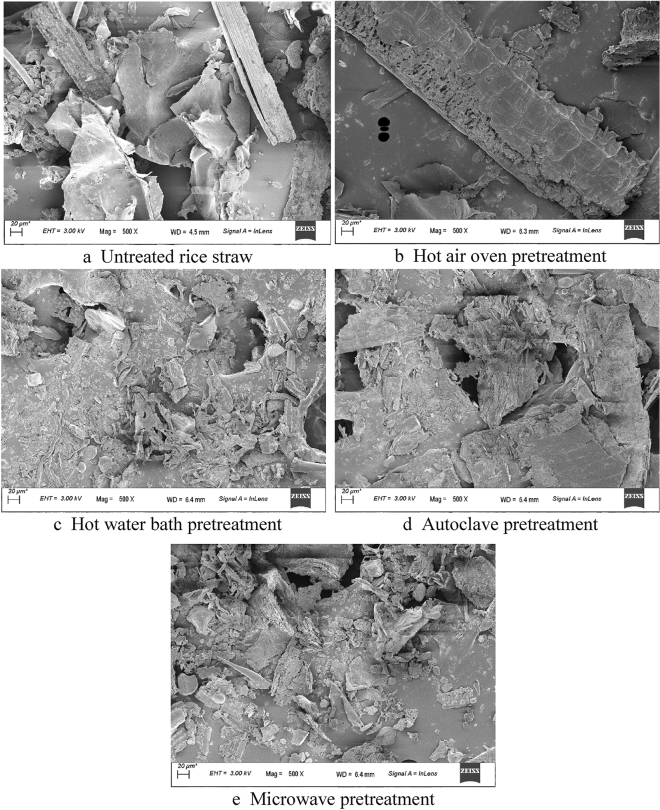
FESEM images of rice straw after different pretreatment methods.

The structural morphology of the untreated sample of RS was well defined (Fig. 3a), which demonstrated the structure's regular, complex, crystalline, and stiff nature. As opposed to Fig. 3b, which illustrated the effects of hot air oven pretreatment through the ruptured and amorphous structure of pretreated RS, it can be noticed that the cellulose and hemicellulose part are largely exposed due to the destruction of the lignin layer. Figure 3c) showed that the RS sample had a more chiselled contour due to hot water bath pretreatment, which enhanced the RS surface area. Figure 3d) showed the RS structure after autoclave pretreatment in a more fractured and damaged state, creating a more accessible region for microbial assault. Figure 3e) depicted the outcome of the microwave pretreatment on the RS sample and suggested that at higher temperatures, the sample would collapse totally and undergo a severe structural breakdown. The structural morphology of the untreated and pretreated samples of RS may therefore be clearly distinguished.

##### FTIR

**Figure 4:**
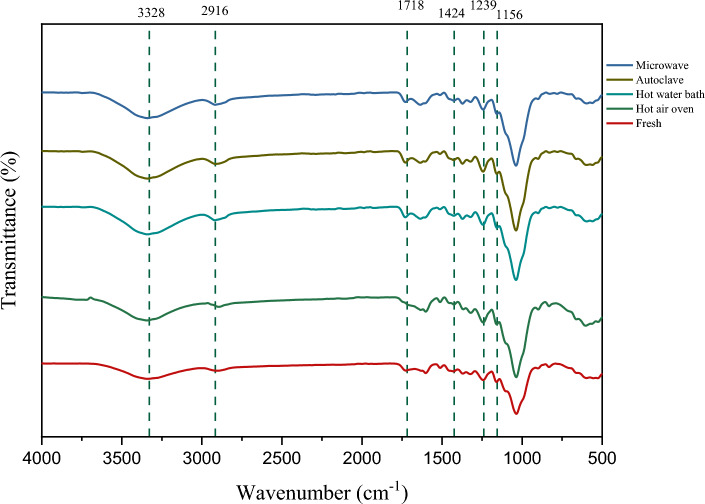
FTIR spectra of rice straw after thermal pretreatment.

Fourier transform infrared (FTIR) analysis was observed on both raw and pretreated RS (Fig. 4). Notably, a reduction in peak intensity at 1156 cm^−1^ (related to C–O–C vibration in cellulose and hemicellulose)^41^ and 1239 cm^-1^ (related to C-O stretching of aryl group) was detected post-pretreatment^42^. A reduction in the peak intensity at 1424 cm^−1^, associated with crystalline cellulose, was also detected^43^. The decline in peak intensity is a result of a decrease in the quantity of crystalline cellulose^44^. The peak at 1718 cm^−1^ representing the carbonyl group^45^ was also seen to decrease in intensity. Furthermore, the peaks at 1723 cm^−1^ and 2916 cm^-1^ displayed a decrease in intensity, possibly due to the alteration or elimination of hemicellulose^44,46^. Notably, peaks at 3328 cm^−1^ were observed, which are due to the O–H stretching of lignin and hydrogen-bonded O–H groups^47^. The decrease in peak intensity at 3328 cm^−1^ was more pronounced in the hot air oven treatment method, indicating a greater removal of lignin.

### Physical characterization of pellets

**Figure 5:**
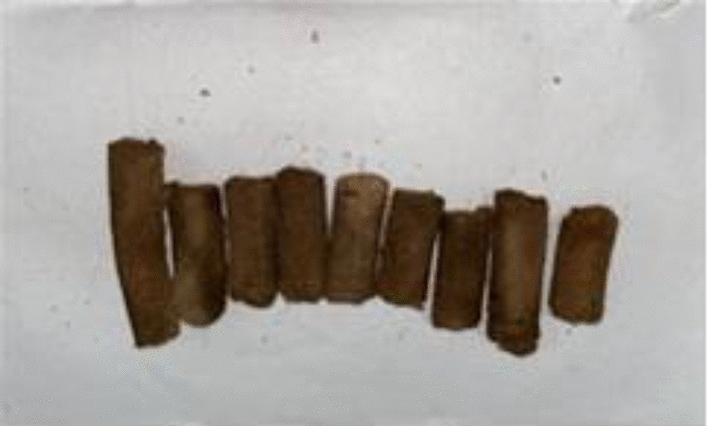
Rice straw and cow dung pellets (RCP).

A comprehensive examination of the pellets’ (Fig. 5) physical characteristics was conducted to assess their suitability for transportation and storage. Three key characterizations were performed: bulk density, water penetration, and friability. Each of these factors plays a critical role in assessing the feasibility and performance of the pellets in practical applications.

#### Bulk density

Bulk density is a crucial parameter for assessing volume reduction and densification. By calculating the bulk density, the quality of the pellets in terms of density, durability, and structural integrity was assessed.

The highest bulk density values for rice straw and cow dung pellets (RCP) were 581.73 kg/m^3^ for the F/M 2.5 composition, as shown in Table 4. These values were found to be significantly higher compared with the bulk density of raw rice straw (around 25 times). In comparison with previous studies, the bulk density of rice straw and pig manure pellets was in the range of 448.5 ± 31.4 kg/m^3^, and sorghum straw and pig manure pellets were measured to be 439.5 ± 13.6 kg/m^3^ ^20^. A different study found that the bulk densities were in the 365–500 kg/m^3^ range for pelletized wheat straw, corn stover, big bluestem, and sorghum stalk^48^. This study's findings emphasize that a higher feed-to-microorganism (F/M) ratio composition led to greater bulk density, indicating successful volume reduction and densification. Additionally, it was observed that cow dung acted as an effective binder, contributing to improved mechanical strength during the pelletization process. The bulk density of crop residue pellets is typically higher than that of raw crop residues due to the compaction and densification during pellet manufacturing. The pellet bulk density can also vary depending on factors such as the pelletization process, pellet size, binder content (if used), and the specific crop residue being pelletized. Higher bulk density in crop residue pellets offers advantages during transportation and storage. It allows for efficient utilization of storage space, reduces transportation costs by maximizing payload, and provides better stability during handling and transport.

**Table 4:** Physical properties of rice straw and cow dung pellets (RCP).

F/M ratio	Bulk density (kg/m^3^)	Water penetration (%)	Friability (%)
F/M 0.5	494.26 ± 37.3	20.26 ± 1.5	25.69 ± 2.6
F/M 1.0	512.19 ± 46.1	18.44 ± 1.3	19.63 ± 2.1
F/M 1.5	533.09 ± 43.8	16.48 ± 0.9	13.42 ± 1.4
F/M 2.0	558.45 ± 48.7	13.28 ± 1.1	8.57 ± 0.7
F/M 2.5	581.73 ± 40.5	7.79 ± 0.4	6.39 ± 0.5

± standard deviation.

#### Water penetration

Water penetration can affect the physical characteristics of crop residue pellets, including integrity, density, swelling, moisture content, and durability. It is important to consider pellets' water resistance and penetration characteristics to ensure their suitability for specific applications and to maintain quality during transportation and storage^49^. Excessive water penetration can compromise the integrity of the pellets. If the pellets are not adequately water-resistant, it can absorb water, become soft, or disintegrate. It can affect the density of the pellets. If the pellets absorb water, it can become heavier, increasing its bulk density. This increased density can impact transportation capacity and may require storage or handling procedure adjustments. If pellets absorb water and then dry out repeatedly, it can lead to cycles of swelling and drying, causing physical stress on the pellet structure, which results in reduced pellet durability. Furthermore, the TS content of the pellets is also lowered with water penetration. Reduced bacterial growth and fermentation due to increased TS content increase shelf life^50^.

The results indicate that different feed-to-microorganism (F/M) ratios influence the water penetration of these pellets. For RCP, the analysis reveals that the F/M 2.5 composition demonstrated the lowest water penetration, followed by F/M 2.0. This suggests that higher F/M ratios, such as F/M 2.5, correspond to decreased water penetration ability in these pellet compositions. These findings emphasize the significance of the F/M ratio in determining the water penetration characteristics of crop residue pellets. Higher F/M ratios, such as F/M 2.5, tend to contribute to reduced water penetration in RCP.

#### Friability test (drop shatter test)

The results of the drop-shatter test indicate the pellet's resistance to mechanical stress and impact. Pellets that exhibit minimal breakage or fragmentation during the test are considered to have higher durability and better resistance to shattering. Conversely, pellets that break easily or show a high degree of fragmentation are considered to have lower durability and poorer resistance to breakage.

The analysis presented in Table 4 focuses on the friability characteristics of different pellet compositions, namely rice straw and cow dung pellets (RCP). The findings indicate that higher feed-to-microorganism (F/M) ratios, specifically the F/M 2.5 composition, resulted in the lowest friability. These results suggest that pellets with higher F/M ratios are more resistant to breakage during loading, unloading, and transportation processes. Lower friability indicates increased pellet durability and decreased susceptibility to mechanical stress, reducing the risk of pellet fragmentation, crumbling, or disintegration during handling and transportation^49^.

#### Best physical characterization of pellets

The physical analysis of crop residue pellets demonstrated the positive impact of higher feed-to-microorganism (F/M) ratios. Pellets with increased F/M ratios exhibited improved qualities in terms of handling, transportation, and storage. It displayed enhanced resistance to moisture absorption, which played a vital role in overall durability and stability. The optimal range of F/M ratios, identified as F/M 2.0 to F/M 2.5, was found to strike a balance between efficient utilization and maintaining the pellets’ structural integrity throughout its lifespan.

### Biochemical methane potential (BMP) study for optimizing total solid content

Maximizing biogas production and achieving process stability rely on the optimization of the total solid content. This optimization offers several advantages, including increased flexibility in feedstock utilization, maintenance of nutrient balance, and improved economic efficiency. By finding the optimal total solid content, a balance is struck between substrate concentration and microbial activity. This balance leads to higher biogas yields and efficient conversion of organic matter. It also prevents process inhibition caused by excessively high solids or low biogas production rates resulting from low solids. Also, optimizing the total solid content fosters stable microbial communities and enables the successful integration of diverse feedstocks. Additionally, this optimization ensures a proper nutrient balance for microbial growth and activity, resulting in enhanced biogas production and improved economic viability of biogas systems.

**Figure 6:**
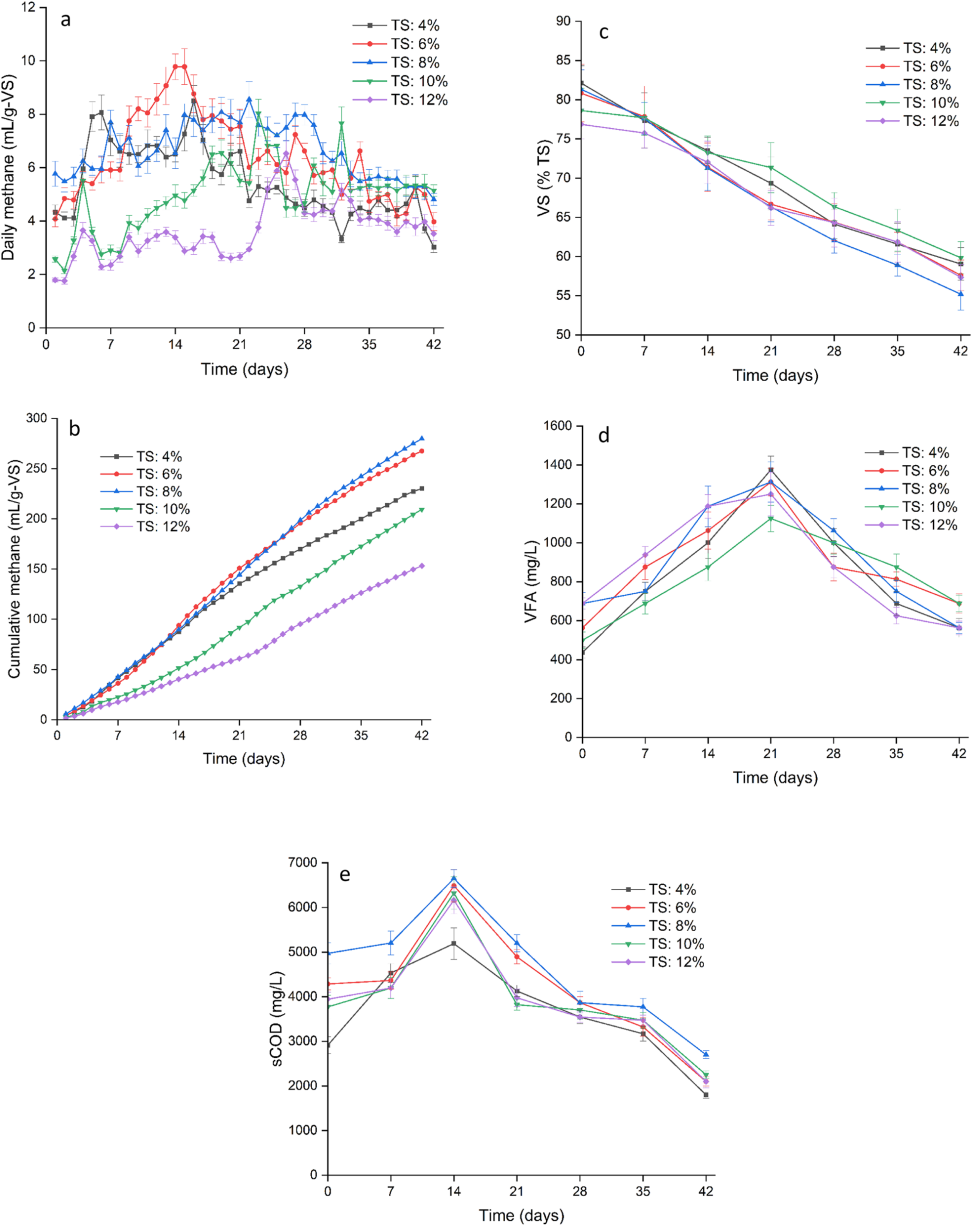
Variation of various parameters with respect to time. **a** Daily methane potential. **b** Cumulative methane production. **c** Volatile solids (%TS) reduction. **d** Volatile Fatty Acids (VFA). **e** Soluble Chemical Oxygen Demand (sCOD).

The methane production from RCP (rice straw and cow dung pellets) was analyzed daily, as shown in Fig. 6a. Specifically, RCP exhibited its highest biogas production (9.78 L/kg/VS_consumed_) on the 15th day at a TS content of 6%. The cumulative methane production increased with an increase in TS from 4 to 8%; and above 8% it started decreasing. It was found to be highest at a TS content of 8% for RCP (279 L/kg-VS_consumed_), as shown in Fig. 6b. The TS content directly affects the substrate concentration and microbial activity within the anaerobic digester. An optimal TS content provides a balance between the availability of organic matter for microbial digestion and the inhibition caused by excessively high or low TS levels. This balance allows for efficient degradation of organic matter and subsequent methane production. A higher percentage of total solids (TS%) in the medium results in a greater contribution to the organic content, thereby leading to a higher concentration of protein and carbohydrate matter within the digester. However, when an overload of organics occurs, this can decrease biogas production above 8% TS. It could be attributed to hindrances in mass transfer and mixing limitations at higher TS content^51^. A similar finding was reported by An et al.^52^, who noted an initial increase in biogas yield with an increase in TS% from 2 to 8%, followed by a decline in yield with further increases in TS%.

The total solids (TS) content in a substrate can significantly impact the degradation of volatile solids (VS) during anaerobic digestion. The VS content represents the organic fraction of the substrate that is biodegradable and can be converted into biogas, primarily methane. If the TS content is too low, the substrate may become diluted, resulting in lower microbial activity and reduced VS degradation. On the other hand, if the TS content is too high, the substrate may become too thick or viscous, hindering the diffusion of microbial enzymes and reducing the degradation efficiency. Finding the appropriate TS content ensures a balance between substrate concentration and microbial activity, leading to optimal VS degradation. The highest VS reduction was observed at specific TS content levels: 8% for RCP (32.1% reduction), as depicted in Fig. 6c. Since VS removal is directly related to biogas yield at varying TS^53^, it decreased over 9% TS. Higher medium TS concentrations lowered VS removal and biogas production^54^. Sentürk et al.^55^ found that substrates with high VS percent had more organic matter and nutrients, which increased biogas yield. However, a larger VS percent increases VFA production, which might induce process failure due to VFA accumulation. However, in our study, we found that the VFA was lower at higher TS (Fig. 6d). It could be attributed to improper mixing and limited microbial access to the viscous substrates at higher TS^51^. This leads to the accumulation of digested products at certain locations or even incomplete digestion. It can be seen from Fig. 6e that the sCOD of the higher solid content, TS 10% and 12%, is comparatively lower than the others (except for TS 4%). This low sCOD concentration subsequently leads to a low amount of VFA production, as was noted.

The impact of TS content on VFA production and accumulation varies depending on substrate characteristics, microbial community composition, and anaerobic digestion parameters. Optimal TS content provides the necessary nutrients and moisture for efficient VFA-to-methane conversion. The study observed the highest VFA reduction at specific TS content levels: 4% for RCP (1375 mL) on the 21st day as shown in Fig. 6d. As time elapsed, sCOD exhibited an increase for all ratios. The VFA amplification amplified sCOD (Fig. 6d,e). The VFA came from carbohydrate hydrolysis. The VFA and sCOD increased together and decreased after peaking. The diverse methanogenic bacteria could convert more soluble organic materials into biogas as sCOD increased. For every TS content, sCOD and VFA were highest on day 14 and 21 respectively. The acidogenic bacterial action, in the beginning, led to a rise in the concentration of VFA, and the start of the methanogenic phase caused a decline in VFA concentration. The TS of 8% had an sCOD of 6648 mg/L, which is the highest among all. Dynamic bacteria in the digester exhausted simple soluble organic matter, lowering sCOD after 14 days. The highest sCOD values were observed on the 14th day for RCP (6648 mg/L) at TS content levels of 8%. In the present study, the VS% (as a percentage of TS) ranged from 76 to 82%, and VS removal (32%) was the highest for the 8% TS mixture, followed by 28% VS removal in the 6% TS mix. The highest cumulative biogas was observed in the mixture with 8% TS. Based on the results, the TS percentage of 8% was found to be best for the anaerobically digesting RCP.

## Practical application and future scope

Agricultural residue and cow dung biomasses are readily accessible and inexpensive. Co-densification of such biomasses offers a practical route to successfully and consistently manufacturing biogas at the commercial level. However, it must be noted that the cost of electricity will increase due to densification. At the same time, the feedstock preparation cost, labour cost, storage cost, and fire protection investment will be reduced^8^. Co-densification has also increased the residue storage time without compromising the biomethane yield^20^. It is recommended to employ the study in large-size biogas digesters and perform a life cycle assessment to understand the environmental benefits this route may have. In addition, optimization of parameters such as mixing rate, temperature, and hydraulic retention time could be performed. Furthermore, more studies on the co-densification of other agricultural residues (wheat straw, sugarcane bagasse) and animal waste (pig dung) should be conducted. Studies on pre-treatment after the pelletization process may also be conducted.

## Conclusion

The present study demonstrates the potential of rice straw and cow dung pellets (RCP) for biogas generation. Thermal pretreatment significantly improved rice straw solubilization and degradability, with hot air oven pretreatment emerging as the most effective method. The pelletization process was effective in mitigating the low bulk density of rice straw, resulting in pellets with significantly higher bulk densities than raw crop residues. The RCP had a bulk density of 581.73 kg/m^3^. The high food-to-microorganism (F/M) ratios have shown favourable physical properties, making the pellets more suitable for handling, transportation, and storage.

Furthermore, the study underscores the importance of the optimizing of total solids (TS) for maximizing methane production. The study found that the combination of specific TS content and feed dilution ratios (FDR) resulted in efficient conversion of complex organic matter and subsequent high methane production. The optimal TS content for RCP was 8% (FDR 1:12). Establishing standard operating procedures for crop residue pellet utilization and improving anaerobic digestion system performance relies on optimizing TS content.

## Data Availability

The data that support the findings of this study are available from the corresponding author upon reasonable request.

## Abbreviations

RS: Rice straw
CD: Cow dung
RCP: Rice straw and cow dung pellets
F/M: Food-to-microorganism ratio
BMP: Biochemical methane potential
TS: Total solids
VS: Volatile solids
sCOD: Soluble chemical oxygen demand
VFA: Volatile fatty acids
